# Chemotherapy Plus Cetuximab versus Chemotherapy Alone for Patients with KRAS Wild Type Unresectable Liver-Confined Metastases Colorectal Cancer: An Updated Meta-Analysis of RCTs

**DOI:** 10.1155/2017/8464905

**Published:** 2017-01-11

**Authors:** W. Lv, G. Q. Zhang, A. Jiao, B. C. Zhao, Y. Shi, B. M. Chen, J. L. Zhang

**Affiliations:** ^1^Department of Hepatobiliary and Transplantation Surgery, The First Hospital of China Medical University, Shenyang, Liaoning Province, China; ^2^Department of Clinical Medicine, First Affiliated Hospital of Zhengzhou University, Zhengzhou, Henan Province, China

## Abstract

*Purpose.* Our study analyses clinical trials and evaluates the efficacy of adding cetuximab in systematic chemotherapy for unresectable colorectal cancer liver-confined metastases patients.* Materials and Methods.* Search EMBASE, PubMed, and the Cochrane Central Register of Controlled Trials for RCTs comparing chemotherapy plus cetuximab with chemotherapy alone for KRAS wild type patients with colorectal cancer liver metastases (CRLMs). We calculated the relative risks (RRs) with 95% confidence interval and performed meta-analysis of hazard ratios (HRs) for the R0 resection rate, the overall response rate (ORR), the progression-free survival (PFS) and overall survival (OS).* Results.* 1173 articles were retrieved and 4 RCTs were available for our study. The four studies involved 504 KRAS wild type patients with CRLMs. The addition of cetuximab significantly improved all the 4 outcomes: the R0 resection rate (RR 2.03, *p* = 0.004), the ORR (RR 1.76, *p* < 0.00001), PFS (HR 0.63, *p* < 0.0001), and also OS (HR 0.74, *p* = 0.04); the last outcome is quite different from the conclusion published before.* Conclusions.* Although the number of patients analysed was limited, we found that the addition of cetuximab significantly improves the outcomes in KRAS wild type patients with unresectable colorectal cancer liver-confined metastases. Cetuximab combined with systematic chemotherapy perhaps suggests a promising choice for KRAS wild type patients with unresectable liver metastases.

## 1. Introduction

Liver is well known as the most common site of colorectal cancer metastasis. Liver metastases have already been found in about 25% patients when establishing the diagnosis of colorectal cancer [1]. Colorectal cancer liver metastasis now has already become a focused point for the researchers recently. Surgery is an effective measure to improve survival rate for patients with resectable metastases. Unfortunately, only about 10% patients with colorectal cancer liver metastases (CRLMs) are accessible to get a surgery treatment at the time of diagnosis [2], and at least two-thirds of the rest of 90% patients died for the reason of unresectable CRLMs (5-year survival rate is almost zero) [3].

During the past decade, the median survival of patients with CRLMs increased quite significantly by systematic chemotherapy [4]. In addition, the median survival has also been improved from 6–8 months to over 20 months by the use of targeted therapy [5]. Recently, the epidermal growth factor receptor (EGFR) has become a promising target for it is activated in colorectal tumors [6]. Inhibition of the active target seems to be a potential choice for patients with CRLMs. For this reason, cetuximab, a strong EGFR inhibitor, has already been focused on the treatment of CRLMs. KRAS is an effector gene in the downstream of EGFR, a paper reported that patients with KRAS mutant type could not benefit from adjuvant chemotherapy and were not sensitive to EGFR inhibitor, and cetuximab is also not effective to KRAS mutant type patients with CRLMs [7, 8].

However, a lot of papers revealed the efficacy of anti-EGFR plus chemotherapy treatment for patients with CRLMs, and four RCT studies have already been published before 2011 [9–12], and even a meta-analysis has been published in 2012 [13] showing a higher level of evidence-based medical evidence on the benefit and disadvantages using anti-EGFR agents in combination with chemotherapy treatment for patients with colorectal cancer, but there are still some controversial issues such as whether cetuximab increases overall survival (OS) or not. According to a new meta-analysis published in 2016, cetuximab does increase the OS of patients with unresectable metastases colorectal cancer [14]. But this study failed to mention the results of patients with colorectal cancer liver-limited metastases. In addition, a randomized controlled trial published in 2013 gave the conclusion that cetuximab benefits the OS of patients with colorectal cancer liver-limited metastases [15], and both the conclusions imply that the conclusion of the meta-analysis studied on the patients only with colorectal cancer liver-limited metastases published in 2012 may be a little unreasonable.

Therefore in this article we perform a meta-analysis of RCTs comparing cetuximab plus chemotherapy with chemotherapy alone with the aim of identifying whether cetuximab plus chemotherapy improves the outcomes of R0 resection rate, overall response rate, progression-free survival, and overall survival of KRAS wild type patients only with colorectal cancer liver-limited metastases or not at a higher level of evidence-based medical evidence.

## 2. Materials and Methods

### 2.1. Search Strategy

A search of PubMed, EMBASE, and Cochrane Library databases (all databases from January 2004 to July 2016) was performed to extract the relevant literature that reports R0 section rate, overall response rate, and outcome on progression-free survival and overall survival of patients with liver-limited metastases which originated from KRAS wild type colorectal cancer and are treated by chemotherapy with or without cetuximab in a randomized controlled trial (RCT). Search terms were as follows: “colorectal cancer metastases” (or “carcinoma” or “malignant tumor”) and “cetuximab”. The latest search was executed on July 13, 2016 and had no limit for language. We start the search from January 2004 because the cetuximab for the treatment of patients with advanced colon cancer was approved by FDA in 2004 [16]. Meanwhile, we included the conference literature as well.

#### 2.1.1. Types of Studies

Only randomized controlled trials (RCTs) provided the outcomes of KRAS wild type colorectal cancer liver-confined metastases patients that were included in the meta-analysis for ensuring the study level. Other nonrandomized trials were all excluded.

#### 2.1.2. Characteristics of Patients Included

The inclusion criteria of patients were as follows:

1. Patients should have been given a confirmed diagnosis of metastatic and liver-limited colorectal cancer (extrahepatic resection must be excluded) and have not received any primary treatments of the metastases till the trial began.
2. Patients included must be KRAS wild type.
3. The liver-limited metastases must be unresectable (according to the definition of single participant).
4. All the patients who did not meet the above criteria should be excluded.

#### 2.1.3. Types of Intervention

Patients who met the criteria (1)–(3) were randomly assigned to chemotherapy alone group or chemotherapy plus cetuximab group in each included study.

#### 2.1.4. The Measurement of Outcomes

The radical resection (R0 resection) rate of liver-confined metastases was the first outcome we measure, and overall response rate (ORR), progression-free survival (PFS), and overall survival (OS) would also be measured in turn.

### 2.2. Data Selection, Extraction, and Analysis

#### 2.2.1. Selection of Studies

This job was executed by two authors (W. Lv and G. Q. Zhang) independently abiding by the above inclusion criteria. Studies would be chosen if they contained the following items:

1. Total population of KRAS wild type patients with liver-limited metastases colorectal cancer
2. The number of R0 resection in the group
3. Either the number of responses or relative risk (RR) (if available)
4. Either PFS months or hazard ratio (HR) (if available)
5. Either OS months or HR (if available)

Once a study contained the 1st and 2nd items, it also contained any of the items of the 3rd to 5th items, and the study would be included. Any discrepancies between the authors were resolved through discussion, rechecking the article content until the authors reached a consensus.

#### 2.2.2. Data Extraction

The data were extracted as follows: the first author, publication year, region, number of patients in each arm, treatments, R0 resection rate (and RR), response rate (and RR), PFS time (and HR), and OS time (and HR).

Three authors (W. Lv, G. Q. Zhang, and A. Jiao) extracted the data independently by the items described above. HRs and their 95% confidence interval (CI) for PFS and OS (if available) were obtained from each primary study. The events of total R0 resections and responses were directly extracted from the studies included or obtained by calculating through the percentages provided by each study included. The proportion of patients with the R0 resection and response outcomes and 95% CIs has been calculated and presented as well as RRs.

#### 2.2.3. Quality Assessment

Two authors (W. Lv and G. Q. Zhang) assessed the quality of the included trials using the quality checklist recommended by the Cochrane Handbook [17]. The following domains on the risk of bias were assessed: randomization, patients blinding, concealed allocation, intention-to-treat analysis, and completeness to follow-up. We resolved all disagreements by discussion and referral to a third author (A. Jiao) for adjudication.

#### 2.2.4. Statistical Analysis

HRs and RRs were both performed in our meta-analysis, and we used Cochran's* Q* test to evaluate the statistical heterogeneity among the studies which had been included in our meta-analysis, and *I*^2^ statistic and *p* value were both used to evaluate the statistical heterogeneity. It is considered that *I*^2^ statistic > 50% and *p* < 0.1 represented significant statistical heterogeneity [18]. In our study, there was no statistical heterogeneity presented in our study, so we cited the fixed effect model in our study. At last, we assessed potential publication biases and two tailed *p* < 0.05 would be identified as significant statistical difference [17].

We evaluated the publication bias existing in our meta-analysis or not according to Begg's test and Egger's test, calculated by software Stata/SE 12.0.

Finally, the results of our meta-analysis were reported as forest plots. Statistical analyses were performed with Stata/SE 12.0 and Review Manager 5.3 (Review Manager (RevMan) [Computer program], Version 5.3, Copenhagen: The Nordic Cochrane Centre, The Cochrane Collaboration, 2014).

## 3. Results

### 3.1. Overview of Studies

**Figure 1:**
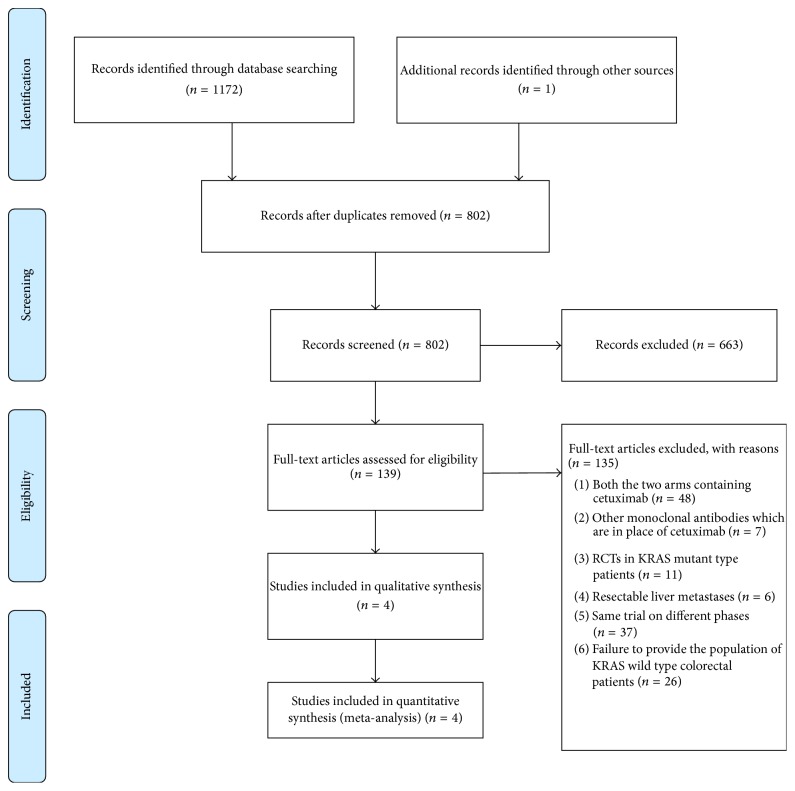
Flowchart of the included studies.

A total of 1173 articles have been retrieved by the search strategy described in Materials and Methods. Most of the studies were excluded only by screening the title for various reasons (Figure 1). There were 139 RCTs left after screening. Furthermore, 135 papers were excluded with the reasons given in Figure 1. Finally, 4 papers (data extracted from 2 published articles and 1 conference abstract that pooled the analysis of another 2 published trials) were considered eligible for inclusion [10–12, 15, 19]. These 4 papers were all RCTs. All trials included chemotherapy plus C arms and chemotherapy alone arms. Characteristics of these studies and the summary of the outcomes had been represented in Table 1, and 504 patients (250 in experimental arms and 254 in control arms) were enrolled in the 4 RCTs. Unresectability criteria were according to the definition of single participant because they were not clearly described.

### 3.2. Quality Assessment

We evaluated the quality of each trial according to five domains: randomization, patients blinding, concealed allocation, intention-to-treat analysis, and completeness to follow-up (Table 2). All included articles described their study design as prospective randomized controlled trials. No studies reported that patient blinding and concealed allocation clearly and all the studies included used intention-to-treat analysis. All the follow-up of the studies has been finished, and all studies had greatly adequate follow-up durations.

### 3.3. Effect of Interventions

#### 3.3.1. R0 Resection Rate

**Figure 2:**
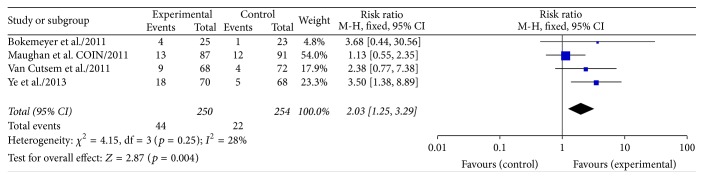
Meta-analysis R0 resection comparing chemotherapy ± cetuximab in patients with liver-limited metastases.

Data on R0 resection rates in KRAS wild type colorectal cancer patients with liver-confined metastases were available in all RCTs [10–12, 15] (504 patients). A fixed effect model has been chosen because the heterogeneity was 28% (*p* = 0.25). The results of our meta-analysis showed that the rate of radical resection of liver metastases was significantly increased from 8.7% to 17.6% by the use of cetuximab (RR 2.03, 95% CI 1.25–3.29; *p* = 0.004; Figure 2).

#### 3.3.2. Response Rate

**Figure 3:**
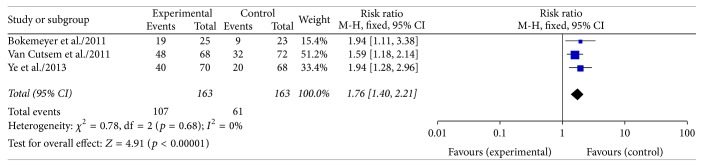
Meta-analysis response rate comparing chemotherapy ± cetuximab in patients with liver-limited metastases.

Data on response rates in KRAS wild type colorectal cancer patients with liver-confined metastases were available in 3 RCTs [10, 12, 15] (326 patients). A fixed effect model has been chosen because the heterogeneity was 0% (*p* = 0.68). The results of our meta-analysis showed that the likelihood of response of the liver metastases was significantly increased from 37.4% to 65.6% by the use of cetuximab (RR 1.76, 95% CI 1.40–2.21; *p* < 0.00001; Figure 3).

#### 3.3.3. Progression-Free Survival

**Figure 4:**
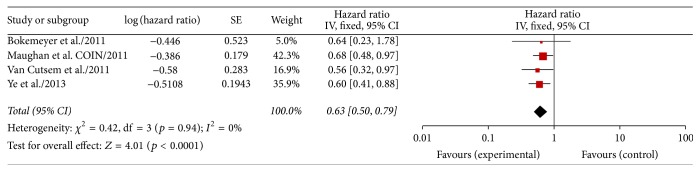
Meta-analysis PFS comparing chemotherapy ± cetuximab in patients with liver-limited metastases.

Data on progression-free survival in KRAS wild type colorectal cancer patients with liver-confined metastases were available in all RCTs [10–12, 15] (504 patients). A fixed effect model has been chosen because the heterogeneity was 0% (*p* = 0.94). The results of our meta-analysis showed that the risk of progression was significantly reduced by the use of cetuximab (HR 0.63, 95% CI 0.50–0.79; *p* < 0.0001; Figure 4).

#### 3.3.4. Overall Survival

**Figure 5:**
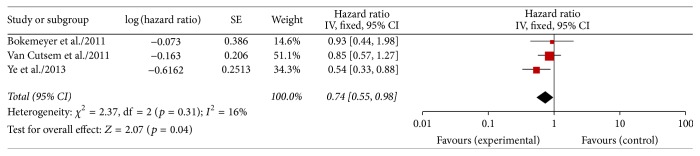
Meta-analysis OS comparing chemotherapy ± cetuximab in patients with liver-limited metastases.

Data on the HRs for death in KRAS wild type colorectal cancer patients with liver-confined metastases were available in 3 RCTs [10, 12, 15] (326 patients). A fixed effect model has been chosen because the heterogeneity was 16% (*p* = 0.31). The results of our meta-analysis showed that the risk of death was significantly reduced by the use of cetuximab (HR 0.74, 95% CI 0.55–0.98; *p* = 0.04; Figure 5), while this outcome is quite different from the conclusion given by the previous studies.

### 3.4. Risk of Bias in the 4 RCTs

According to Begg's test (*p* = 0.734) and Egger's test (*p* = 0.680), we could give the conclusion that publication bias did not exist in our meta-analysis.

## 4. Discussion

The liver is the most common metastatic site of colorectal cancer, and the resection of liver metastases usually has a significant impact on the prognosis [20]. Systematic chemotherapy had already been regarded as an effective way to shrink the size of liver metastases for resection. Some studies reported that systematic chemotherapy does have credible ability to reduce the tumor size and has made a few patients with unresectable liver metastases undergo hepatic resection after chemotherapy treatment (12.5%, 3.3%) [2, 21]. But the rate is still not high enough.

In order to identify the effect of the addition of cetuximab more systematically we performed our meta-analysis for a new RCT has been published. In our study, we described the outcomes of adding cetuximab in systematic chemotherapy and showed R0 resection rate, response rate, and PFS of KRAS wild type patients with CRLMs benefited from it. However, importantly, we also found that OS of KRAS wild type patients with CRLMs can also benefit from adding cetuximab; this result is quite different from the research published before, suggesting that cetuximab may be helpful for improving OS of KRAS wild type patients with CRLMs. In addition, the R0 resection rate is also higher than the results published before [13] (8.7%–17.6%, RR 2.03, *p* = 0.004 versus 11%–18%, RR 1.59, *p* = 0.04).

The reasons for such significant differences between Petrelli and Barni's study and our study perhaps ascribe to the 3 following reasons:

(1st) The studies included in each meta-analysis are different. The COIN trial, the OPUS study, and the CRYSTAL trial are the 3 RCTs included in both Petrelli and Barni's and our meta-analyses; however, our study did not include the RCT performed by Douillard et al. because this RCT mixed cetuximab and panitumumab in their study, while Petrelli and Barni's study is included. There are concerns that a lot of patients in the COIN trial go through reducing the drug dose in the period of treatment because of adverse events, so perhaps the patients in COIN trial had not gotten a full therapeutic benefit. Meanwhile, the RCT performed by Ye et al. (published in 2013, after Petrelli and Barni's study) did not reduce the drug dose in order to compromise on adverse events. So the full therapeutic benefit may not have been realized. Our study indicated adding cetuximab to potentially improve the overall survival rate.

(2nd) The drugs used in each meta-analysis are different. Petrelli and Barni's meta-analysis includes the RCT performed by Douillard et al. which mixed cetuximab and panitumumab in their study. To our knowledge, there is still not a RCT for comparing cetuximab with panitumumab, but the conclusion that panitumumab is not equally efficacious against the disease has been already reported [22]. Meanwhile, cetuximab can cause antibody dependent cellular cytotoxicity (ADCC) against tumor, but panitumumab does not have such effect because cetuximab is an IgG1 class antibody but panitumumab is an IgG2 class antibody [23].

(3rd) Racial differences existed between Petrelli and Barni's and our meta-analyses. All the patients included in Petrelli and Barni's meta-analysis are westerner, but the patients included in our study consist of westerner and Chinese. The racial differences perhaps lead to the different results between Petrelli and Barni's and our meta-analyses. As far as we know, there is not a credible evidence performed to prove that anti-EGFR does have the equal efficacy on different races yet.

However, these discussions and conclusions should be interpreted with caution due to the small sample size.

Although our meta-analysis reveals some new results, however, there are also some limitations in it. First, the number of patients analysed was limited, and the analysis of outcome as a function of KRAS status was performed retrospectively. Second, the unresectable criteria were not clearly described. Third, the patients in the former 3 RCTs included are only a subgroup of all metastatic patients rather than the last fourth RCT which enrolled solely patients with liver-limited metastases. Finally, we failed to obtain all the individual data of patients included as this is a paper-based study.

In summary, despite these defections, our study implies that the addition of cetuximab to systematic chemotherapy confers not only a significant benefit in terms of resectability, PFS, and response rate compared to systematic chemotherapy alone but also a significant benefit in terms of OS for the first time, especially for Chinese. Despite these limitations of this analysis, systematic chemotherapy plus cetuximab seems to be a promising choice for downsizing unresectable liver-confined metastases and prolonging survival time in KRAS wild type patients with CRLMs.

## Figures and Tables

**Table 1:** Characteristics of the RCT studies included in our meta-analysis.

Author year	Number of LCM wt pts (exp/ctr)	Treatments (exp/ctr) arms	R0 resection% (exp/ctr) RR (*p*)	Response rate% (exp/ctr) RR (*p*)	PFS months exp versus ctr/HR (*p*)	OS months exp versus ctr/HR (*p*)
Bokemeyer et al./2011 (OPUS)	48 (25/23)	FOLFOX + C versus FOLFOX	16/4 3.68 (0.23)	76/39 1.94 (0.02)	11.9 versus 7.9/0.64 (0.39)	26.3 versus 23.9/0.93 (0.85)

Van Cutsem et al./2011 (CRYSTAL)	140 (68/72)	FOLFIRI + C versus FOLFIRI	13.2/5.5 2.38 (0.13)	70.5/44.4 1.59 (0.003)	11.8 versus 9.2/0.56 (0.04)	27.8 versus 27.7/0.85 (0.43)

Maughan et al./2011 (COIN)	178 (87/91)	XELOX or FOLFOX + C versus XELOX or FOLFOX	15/13 1.13 (0.74)	NR NR	NR/0.68 (0.03)	NR

Ye et al./2013	138 (70/68)	FOLFOX + C versus FOLFOX	25.7/7.4 3.50 (0.004)	57.1/29.4 1.94 (<0.01)	10.2 versus 5.8/0.60 (0.004)	30.9 versus 21.0/0.54 (0.013)

LCM: liver-confined metastases; RR: relative risk; HR: hazard ratio; PFS: progression-free survival; OS: overall survival; wt: wild type; pts: patients; exp: experimental; ctr: control. FOLFOX refers to folinic acid (FOL) + fluorouracil (F) + oxaliplatin (OX); FOLFIRI refers to folinic acid (FOL) + fluorouracil (F) + irinotecan (IRI); XELOX refers to capecitabine (XEL) plus oxaliplatin (OX); C refers to cetuximab.

**Table 2:** Quality of each RCT included in the meta-analysis.

Author year	Randomization	Patients blinding	Concealed allocation	Intention-to-treat analysis	Completeness to follow-up
Bokemeyer et al./2011 (OPUS)	Yes	Unclear	Unclear	Yes	Yes
Van Cutsem et al./2011 (CRYSTAL)	Yes	Unclear	Unclear	Yes	Yes
Maughan et al./2011 (COIN)	Yes	Unclear	Unclear	Yes	Yes
Ye et al./2013	Yes	Unclear	Unclear	Yes	Yes
